# Pharmacokinetic Evaluation of a Single 5-Gram Bolus of Creatine Monohydrate Versus Two Other Creatine-Containing Investigational Products

**DOI:** 10.7759/cureus.24395

**Published:** 2022-04-22

**Authors:** Jose Antonio, Cassandra Evans, Lia Jiannine, Jason Curtis, Katarzyna Wojnas, Victoria Burgess, Darryn Willoughby, Corbin Hohl, Brent Petersen, Sarah Flynn, Joshua Baisley, Gaurav Parekh, Doug Kalman

**Affiliations:** 1 Health Care Sciences, Nova Southeastern University, Fort Lauderdale, USA; 2 Nutrition, Nova Southeastern University, Fort Lauderdale, USA; 3 Exercise Science, Keiser University, Fort Lauderdale, USA; 4 Psychology and Neuroscience, Nova Southeastern University, Fort Lauderdale, USA; 5 Health and Human Performance, Concordia University, Chicago, USA; 6 Exercise and Sport Science, University of Mary Hardin-Baylor, Belton, USA; 7 Protein Research and Development, Glanbia Nutritionals, Twin Falls, USA; 8 Ingredients Research and Development, Glanbia Nutritionals, Twin Falls, USA; 9 Product Strategy and Technology, Glanbia Nutritionals, Twin Falls, USA; 10 Clinical Design and Delivery, Nutrasource Pharmaceutical and Nutraceutical Services Inc., Guelph, CAN; 11 Research and Development, Glanbia Nutritionals, Twin Falls, USA; 12 Scientific Affairs, Nutrasource Pharmaceutical and Nutraceutical Services Inc., Guelph, CAN

**Keywords:** creatine monohydrate, dietary supplements, pharmacokinetic, creatine, nutrition

## Abstract

The purpose of this study was to determine the relative pharmacokinetics of creatine monohydrate delivered as a formula or as a pure powder (all mixed in solution). A single 5 g bolus of creatine monohydrate was ingested as CreaBev 1, CreaBev 2, or creatine monohydrate. Participants we assigned a test product and monitored in a supervised laboratory setting for ingestion and all blood draws starting 30 min post-ingestion to the 6-h mark. Standard pharmacokinetic analysis was undertaken to determine relative maximum concentration (Cmax), time to maximum concentration (Tmax), and area under the curve (AUC) for the products. Cmax data indicate that CreaBev 1 10.55±4.10, CreaBev 2 15.45±5.48, and creatine monohydrate 12.77±4.0 nmol/h/μL. The Tmax analysis demonstrated CreaBev 1 1.20±1.01, CreaBev 2 1.23±0.65, and creatine monohydrate 0.91±0.2 h. The AUC data indicate that CreaBev 1 22.90±9.17, CreaBev 2 33.92±9.52, and creatine monohydrate 29.58±11.93 nmol/h/μL. When examining the data for pharmacokinetics, the AUC and Cmax pharmacokinetics were greatest for CreaBev 2 (p<0.021 and 0.020). Within the confines of this study, CreaBev 2 produced the highest blood concentrations of creatine as compared to creatine monohydrate and CreaBev 1.

## Introduction

Creatine is a naturally occurring compound that is found predominantly in skeletal muscle [1-6]. There is a plethora of evidence that creatine supplementation is safe and effective [2]. Powdered or solid forms of creatine are more stable than aqueous solutions containing creatine [1,7-10]. However, it is generally accepted that creatine consumed as a drink has better bioavailability [8,10,11]. Studies have suggested that creatine is completely absorbed due to the absence of creatine found in fecal samples [10,12]. Highly acidic environments can negatively affect the breakdown of creatine [2,7,13]. It has been suggested that creatine is absorbed in the gastrointestinal tract [1,6,10,14,15]. The acidic environment of the stomach could potentially lead to degradation of creatine leading to lower absorption and ultimately less uptake by muscles. There are few studies that have examined the bioavailability and absorption of creatine. For instance, Harris et al. observed plasma concentrations of creatine over 6 h following the consumption of meat or a creatine supplement [11]. Participants ingested steak, creatine in solid form, or creatine in a solution. Blood samples were taken every 30 min for the first 2 h, then every hour for the next 6 h. Creatine administered in a solution resulted in a greater peak plasma concentration followed by a rapid decline while the creatine consumed via steak resulted in a lower initial peak but an extended decline in concentration. This study suggests liquid creatine supplements are more bioavailable than solid or meat products. Thus, the objective of this investigation was to evaluate the pharmacokinetic (PK) response in healthy exercise-trained male participants after the ingestion of a single 5 g bolus of creatine monohydrate in comparison to two investigational creatine-containing products (i.e., CreaBev 1 or CreaBev 2).

## Materials and methods

Participants and sample size

A total of 37 exercise-trained male participants volunteered for this investigation. Participant demographics and training history were collected during baseline via questionnaire. Research participants were randomized in a double-blind, placebo-controlled trial in which they received one of the following treatments: creatine monohydrate (5 g), CreaBev 1 (5 g of creatine), or CreaBev 2 (5 g of creatine). CreaBev 1 and CreaBev 2 were investigational products in powdered form (Table 1 and Table 2, respectively). The products did not contain protein and contained ≤1 g of carbohydrates. All participants signed a written informed consent that was approved by the institutional review board of the university (IRB# 2019-568) prior to conducting any study-related activities.

**Table 1:** CreaBev 1 supplement facts panel. *Percent daily values are based on a 2000-calorie diet. **Daily value is not established. Other ingredients: maltodextrin, soy lecithin, α-cyclodextrin.

Serving size (7 g)	Amount per serving	% Daily value
Calories	10	-
Total fat	1 g	1%*
Carbohydrate	1 g	<1%*
Creatine monohydrate	5 g	-**

**Table 2:** CreaBev 2 supplement facts panel. *Percent daily values are based on a 2000-calorie diet. **Daily value is not established. Other ingredients: soluble pea fiber, sunflower lecithin.

Serving size (7 g)	Amount per serving	% Daily value
Calories	5	-
Carbohydrate	1 g	<1%*
Creatine monohydrate	5 g	-**

Study design

**Figure 1:**
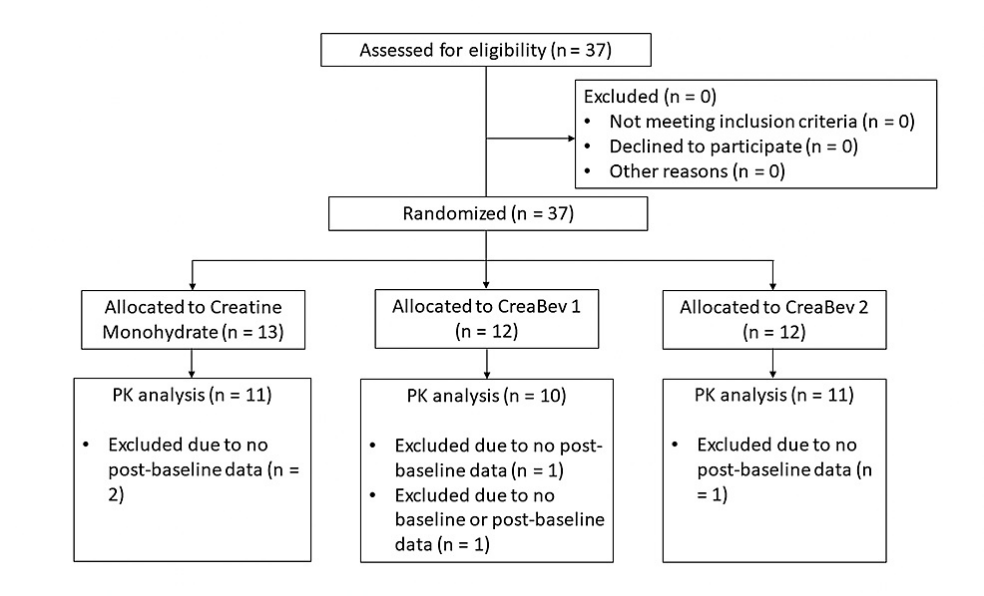
CONSORT diagram of participant disposition. CONSORT: Consolidated Standards of Reporting Trials; PK: pharmacokinetic

**Figure 2:**
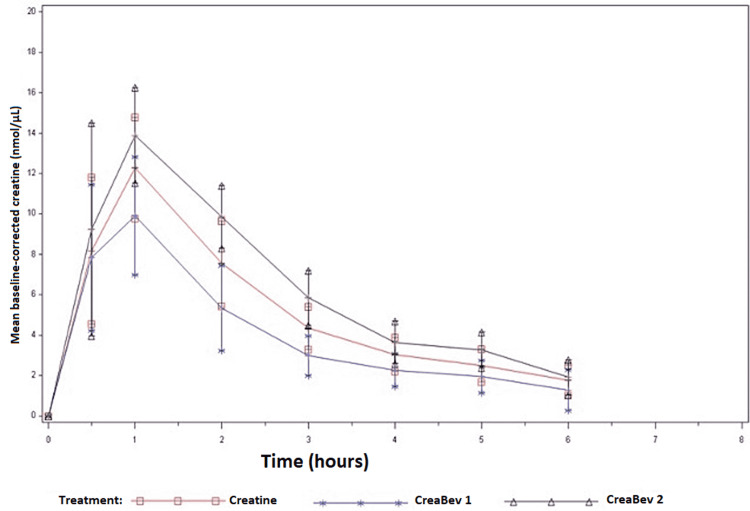
Serum creatine concentrations plotted over a 6 h period.

Each participant arrived at the laboratory under standardized conditions at approximately 8 h. After consenting to participate in the study, the following vitals were ascertained: resting heart rate, resting blood pressure, height, weight, and body composition. Each research participant had their initial blood draw performed. Subsequently, research participants were randomized to consume a 5 g single bolus of either generic creatine monohydrate or CreaBev™ branded creatine (i.e., CreaBev 1 or CreaBev 2; Figures 1, 2). The test products were in powdered form and mixed with ~10 ounces of water and given to each respective study participant. Consequently, the research participants had their blood drawn by a trained phlebotomist at baseline (pre-dose), and again at 0.5 hour (h), 1 h, 2 h, 3 h, 4 h, 5 h, and 6 h post-dose. All blood samples were centrifuged to separate the cells from the plasma. The samples were analyzed for plasma creatine via the method delineated herein [8]. Participants received a pre-dose creatine-free standardized meal (i.e., bagels with cream cheese) after completing the initial (baseline/pre-dose) blood test for serum creatine, and another creatine-free meal (i.e., cheese pizza) between the third and fourth hours post-dose. After the final blood draw at 6 h post-dose, resting heart rate and blood pressure were assessed (Figure 1).

Venipuncture

Blood samples were collected using a sterile venipuncture procedure to place an intravenous (IV) catheter in the antecubital fossa region of each research participant's arm. Non-dominant arms were used, and a second venipuncture procedure was performed with participant consent if the vein collapsed prior to completion of blood collection. Catheters were connected to Luer-Lok (Franklin Lakes, NJ: Becton, Dickinson and Company) access devices to collect blood samples and sterile saline was used to flush the catheter line following each blood sample collection. Blood samples were drawn into 3 mL ethylenediaminetetraacetic acid (EDTA) plasma collection vacutainer tubes. Each participant completed a baseline blood sample collection and then drank a randomly assigned creatine sample. Blood samples were subsequently drawn from the IV catheter at 0.5 h following creatine ingestion, and then at 1 h, 2 h, 3 h, 4 h, 5 h, and 6 h. After each blood sample was collected, tubes were gently inverted eight to 10 times immediately and then placed in a centrifuge to be spun for 12 min at 3000 revolutions per minute (RPM) to separate plasma from blood cells. Plasma samples were then pipetted into plastic test tubes and then placed in a -30°F freezer for storage until shipment for creatine level testing by the contract analytical laboratory (any remaining biological material was then disposed of per standard procedures).

Body composition

Body composition was assessed with a multi-frequency bioelectrical impedance device (InBody® 270; Cerritos, CA: InBody USA) to determine each participant’s physical characteristics. Participants were instructed to arrive at the laboratory after a 3 h fast. Study participants stood on the platform of the device barefoot with the soles of their feet on the electrodes. They then grasped the handles of the unit with their thumb and fingers to maintain direct contact with the electrodes. They stood still for ~1 min while keeping their elbows fully extended and their shoulder joint abducted to about a 30-degree angle. Body composition was assessed at baseline. InBody® 270 is considered a reliable and valid way of assessing body composition [16].

Assessment of serum creatine

Total serum creatine (STC) was determined using a commercially available colorimetric assay kit (Milpitas, CA: BioVision), detected at a wavelength of 570 nm (Machek et al. in 2020) [17]. Serum creatinine (CRT) was determined by commercially available enzyme-linked immunosorbent assay kits (San Diego, CA: MyBioSource), detected at a wavelength of 450 nm. All samples were compared against control peptides of known concentrations in which a standard curve is generated, and sample concentrations determined. Assays involved the use of a microplate reader (X-Mark; Hercules, CA: Bio-Rad) and data reduction software (Microplate Manager; Hercules, CA: Bio-Rad). Samples were run in duplicates. The mean coefficient of variation between all duplicates was 3.53% and the SD was 2.45.

Pharmacokinetic analysis

Standard pharmacokinetic analysis along with appropriate statistical techniques was used to determine the pharmacokinetic profile of the three study products, allowing for comparative analysis regarding the ability to deliver creatine to the body, coupled with the amount of creatine delivered and speed of relative delivery as measured by standard blood levels of creatine. As creatine is an endogenous compound in humans, post-dose concentrations were adjusted by subtracting baseline (pre-dose value) from subsequent values. Five participants were excluded from the analysis they did not have any post-baseline data (i.e., the phlebotomist could not further draw blood). Where blood was not collected due to catheter issues and/or venipuncture difficulties, values were considered missing and were not inputted for pharmacokinetic calculations. The final study population included 32 participants (i.e., 11 received creatine monohydrate, 10 received CreaBev 1, and 11 received CreaBev 2).

Statistical analysis

All data are presented as the mean+SD. With regards to blood creatine concentrations, ANOVA was used to compare differences between groups for area under the curve (AUC) and maximum concentration (Cmax). AUC was calculated using the trapezoidal method using linear interpolation between data points to calculate the AUC. For time to maximum concentration (Tmax), the Kruskal-Wallis test was used to compare the overall difference between groups and comparisons between test products were tested using the Wilcoxon rank-sum test. All statistical analysis was conducted at the alpha level of 0.05 for significance, using SAS (Cary, NC: SAS Institute) version 9.4.

## Results

The characteristics of the study participants (male, n=37) were as follows: age 23±7 years, height 181.3±11.5 cm, body mass 80.2±12.4 kg, % body fat 13.3±5.6, fat mass 10.7±4.8 kg, fat-free mass 68.1±14.7 kg, total years of training 9±7 years, mean hours of aerobic exercise per week 8±7 h, mean hours of resistance training per week 6±4 h. There were significant differences in AUC and Cmax between CreaBev 1 and CreaBev 2; however, there were no differences in AUC, Cmax, or Tmax between the other groups (Table 3, Figure 2).

**Table 3:** Baseline corrected pharmacokinetic parameters - Cmax, Tmax, and AUC for serum creatine. *The only significant differences were for AUC and Cmax between CreaBev 1 and CreaBev 2. All data are expressed as the mean±SD. AUC: area under the curve; Cmax: maximum concentration; Tmax: time to maximum concentration

Baseline corrected pharmacokinetic parameters	Creatine monohydrate (n=11)	CreaBev 1 (n=10)	CreaBev 2 (n=11)	p-Value		
				Creatine vs. CreaBev 1	Creatine vs. CreaBev 2	CreaBev 1 vs. CreaBev 2
Cmax (nmol/µL)	12.77±4.00	10.55±4.10	15.45±5.48	0.277	0.182	0.021*
Tmax (h)	0.91±0.20	1.20±1.01	1.23±0.65	0.776	0.372	0.672
AUC (nmol*h/µL)	29.58±11.83	22.90±9.17	33.92±9.52	0.148	0.329	0.020*

## Discussion

This investigation assessed two different investigational products (i.e., CreaBev 1, CreaBev 2) in comparison to generic creatine monohydrates. The time course of plasma creatine was similar in all three groups. Peak creatine concentrations occurred between 0.9 and 1.2 h post-consumption with no differences between groups. The area under the curve and maximal concentrations of creatine differed only in the CreaBev 1 and CreaBev 2 groups; however, creatine monohydrate did not differ from the two investigational products. In a study measuring serum concentrations of creatine and creatinine, Schedel et al. administered 20 g of creatine to participants and collected blood samples at various time intervals [18]. Two participants (male, n=1) were administered different doses of creatine on separate occasions with a two-week washout period in-between each visit. We observed a rapid initial peak concentration followed by a progressive decline occurring after 3 h. The individual case studies revealed a dose-dependent increase in creatine serum concentrations [17].

The data from our current investigation are similar to Harris et al. [19]. In that investigation, a 5-g dose of creatine resulted in a peak at 1 h post-consumption [19]. Persky et al. found that a single oral dose of creatine monohydrate (71 mg/kg body weight) in six healthy males resulted in a Tmax of 1.9±0.9 h [20]. Moreover, Rawson et al. discovered that Tmax was reached at 1.3±0.1 h and 1.6±0.2 h, respectively in young (24 years) and old (78 years) male participants after the consumption of 5 g of creatine monohydrate; nevertheless, AUC was similar between the older and younger male participants [21]. Jäger et al. examined the effects of isomolar amounts of creatine (4.4 g) in the form of creatine monohydrate, tri-creatine citrate, or creatine pyruvate [20]. Interestingly, they discovered the mean peak concentrations were significantly higher with creatine pyruvate in comparison to creatine monohydrate and tri-creatine citrate; however, there were no differences between creatine monohydrate vs. tri-creatine citrate. Nonetheless, despite the higher plasma concentrations that resulted from creatine pyruvate ingestion, the authors of this investigation suggest that these differences are not likely to have a meaningful physiological difference inasmuch as the absorption of creatine monohydrate is close to 100% [21]. Thus, the time course for elevations in creatine concentration post-consumption seems to be consistent across investigations (i.e., between 1 and 2 h). In the current investigation, one of the investigational products (i.e., CreaBev 2) demonstrated superiority to CreaBev 1; however, in comparison to generic creatine monohydrate, no differences existed. The limitations of this study were the small sample size of each group and the lack of measurement of creatine uptake by the muscle.

## Conclusions

In summary, the absorption rate and extent, as indicated by the mean Cmax and mean plasma AUC, were significantly higher for creatine from CreaBev 2 compared to creatine from CreaBev 1. Although not statistically significant, CreaBev 2 had a higher mean Cmax and AUC compared to creatine monohydrate. The Tmax was on average between 55 and 75 min post-dose for the three formulations. Since creatine monohydrate is virtually all absorbed, it is unknown if the slight differences found in the pharmacokinetics of the different investigational creatine products (i.e., CreaBev 2) would result in meaningful physiological differences.
